# Methylphenidate Decreased the Amount of Glucose Needed by the Brain to Perform a Cognitive Task

**DOI:** 10.1371/journal.pone.0002017

**Published:** 2008-04-16

**Authors:** Nora D. Volkow, Joanna S. Fowler, Gene-Jack Wang, Frank Telang, Jean Logan, Christopher Wong, Jim Ma, Kith Pradhan, Helene Benveniste, James M. Swanson

**Affiliations:** 1 National Institute on Drug Abuse, Bethesda, Maryland, United States of America; 2 National Institute on Alcohol Abuse and Alcoholism, Bethesda, Maryland, United States of America; 3 Medical Department, Brookhaven National Laboratory, Upton, New York, United States of America; 4 Child Development Center, University of California Irvine, Irvine, California, United States of America; Chiba University Center for Forensic Mental Health, Japan

## Abstract

The use of stimulants (methylphenidate and amphetamine) as cognitive enhancers by the general public is increasing and is controversial. It is still unclear how they work or why they improve performance in some individuals but impair it in others. To test the hypothesis that stimulants enhance signal to noise ratio of neuronal activity and thereby reduce cerebral activity by increasing efficiency, we measured the effects of methylphenidate on brain glucose utilization in healthy adults. We measured brain glucose metabolism (using Positron Emission Tomography and 2-deoxy-2[18F]fluoro-D-glucose) in 23 healthy adults who were tested at baseline and while performing an accuracy-controlled cognitive task (numerical calculations) given with and without methylphenidate (20 mg, oral). Sixteen subjects underwent a fourth scan with methylphenidate but without cognitive stimulation. Compared to placebo methylphenidate significantly reduced the amount of glucose utilized by the brain when performing the cognitive task but methylphenidate did not affect brain metabolism when given without cognitive stimulation. Whole brain metabolism when the cognitive task was given with placebo increased 21% whereas with methylphenidate it increased 11% (50% less). This reflected both a decrease in magnitude of activation and in the regions activated by the task. Methylphenidate's reduction of the metabolic increases in regions from the default network (implicated in mind-wandering) was associated with improvement in performance only in subjects who activated these regions when the cognitive task was given with placebo. These results corroborate prior findings that stimulant medications reduced the magnitude of regional activation to a task and in addition document a “focusing” of the activation. This effect may be beneficial when neuronal resources are diverted (i.e., mind-wandering) or impaired (i.e., attention deficit hyperactivity disorder), but it could be detrimental when brain activity is already optimally focused. This would explain why methylphenidate has beneficial effects in some individuals and contexts and detrimental effects in others.

## Introduction

Stimulant medications such as methylphenidate (MP) are used extensively in the treatment of Attention Deficit Hyperactivity Disorder (ADHD) to decrease symptoms of inattention[1] Also, in certain conditions (e.g., sleep deprivation), MP may improve attention and performance of individuals without ADHD[2]. Indeed the past decade has seen an increase in the use of stimulant medications as cognitive enhancers that is of increasing concern both because of their side effects as well as their potential for abuse and addiction[3], [4].

The biochemical mechanisms of action of MP have been well characterized: it increases extracellular levels of dopamine and norepinephrine by blocking the respective monoamine transporters[5]. It is unclear how these actions relate to its effects in attention and performance. Since dopamine and norepinephrine decrease background firing rates of neuronal cells increasing signal-to-noise ratio[6], [7], we hypothesized that in humans MP's dopaminergic and noradrenergic effects by decreasing non-task related activity should reduce the amount of glucose utilized by the brain while performing a cognitive task.

**Figure 1:**
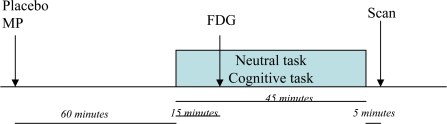
Schematic diagram of experimental procedure. Placebo (PL) or methylphenidate (MP) was given 60 minutes prior to initiation of Cognitive or Neutral tasks, which lasted 45 minutes. [^18^F]FDG was injected 15 minutes after task initiation (75 minutes after MP or PL) and scans were started 35 minutes after injection.

We measured regional brain glucose metabolism in 23 healthy subjects when they performed a mathematical task with difficulty controlled to achieve 80% accuracy. The difficulty-controlled task was performed after administration of placebo and after administration of MP (20 mg, oral), and these conditions were compared to a control condition, which consisted of viewing nature cards with not performance required (non-task condition) after being given a placebo. In addition, 16 of the subjects were tested in a fourth condition (non-task condition after being given MP) (Figure 1).

## Results

PET imaging documented that the cognitive task significantly increased whole brain metabolism when compared with the control condition both when given with placebo and with MP. Whole brain metabolism differed significantly for the conditions (p<0.0005) and was lower for the control (36.6±6 µmol/100 g/min) and the neutral non-task condition preceded by MP (35.8±5 µmol/100 g/min) than for the cognitive task with placebo (43.2±7 µmol/100 g/min) or the cognitive task preceded by MP (40.3±7 µmol/100 g/min). The increase in whole brain metabolism was significantly smaller when the cognitive task was preceded by MP, than when preceded by placebo (11±22% versus 21±26%; p<0.01). Individual analysis of the responses revealed that 16 of the 23 subjects had less activation with MP than with placebo when performing the task, 5 had greater activation with MP than with placebo and 2 did not differ (Chi-Square p<0.02) (Table 1).

**Table 1:** Differences in brain metabolic activation to the task (percent change) when given with MP when compared with placebo (PL) between subjects in whom MP attenuated brain activation versus those in whom it enhanced activation along with their baseline metabolic measures.

	Attenuated Response with MP	Enhanced Response with MP	Difference
Number Ss	16	5	*p<0.02*
% Brain PL v MP	−16±14	+7±1	*p<0.004*
Baseline metabolism	35±4 µmol/100 g/min	39±7 µmol/100 g/min	*p<0.09*

Two subjects showed no differences between MP and placebo (data not included). Comparisons correspond to chi square for the subject numbers (Ss) and to student t-tests (unpaired, two test) for the other comparisons.

Correlation analysis between the differences in brain activation when the task was given with placebo versus MP showed a significant correlation with baseline metabolism (r = 0.48, p<0.05); the lower the metabolism at baseline the greater the attenuation of the activation by the task by MP. The difference in brain activation when the task was given with placebo versus MP was also significantly correlated with the activation to the task with placebo (r = 0.57, p<0.005) but not with activation to the task with MP (r = 0.02, NS) indicating that the response to MP was dependent both on metabolism at baseline and on the level of activation by the task when given without a pharmacological challenge.

There were no differences in money made (surrogate marker of performance) when the cognitive task was done with placebo ($47.60±4) versus when it was done with MP ($48.80±3). However there was significant intersubject variability: 7 subjects made more money with MP, 4 made less money and 12 made the same amount as with placebo. Correlation analysis between the differences in money earned when performing the cognitive task with MP versus placebo and the metabolic differences in activation between these two conditions were significant in paracentral lobule (r = 0.50, p<0.02), superior (r = 0.50, p<0.02) and inferior parietal cortices (r = 0.43, p<0.05), dorsal (r = 0.45, p<0.05) and posterior CG/precuneus (r = 0.44, p<0.05). The greater the attenuation the larger the amount of money made with MP.

**Figure 2:**
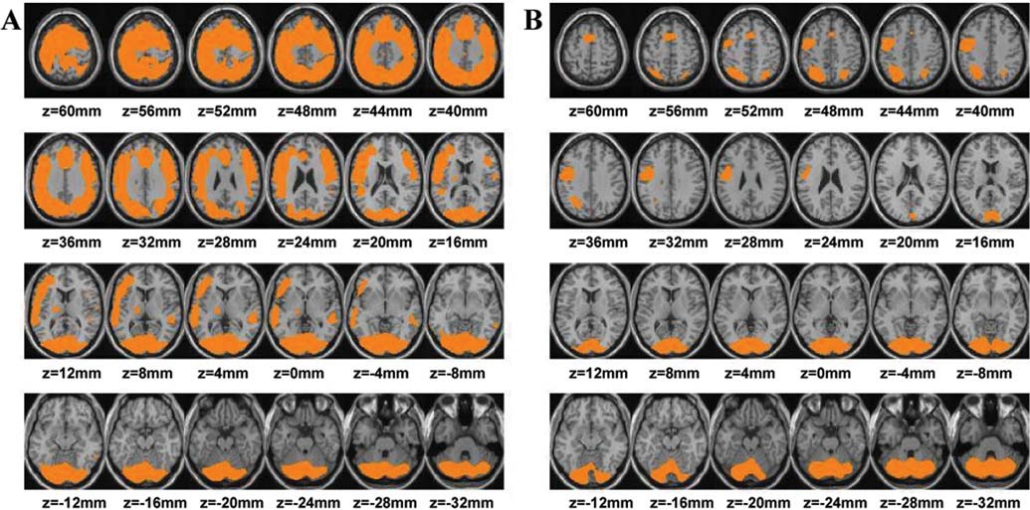
Brain activation with the task after placebo (PL) and after methylphenidate (MP). A. SPM results showing the areas that had increases in metabolism for the cognitive task with placebo versus the control conditions; B. SPM results showing the areas that had increases in metabolism for the cognitive task with MP versus the control conditions. Comparisons correspond to paired t tests (p<0.001 uncorrected >100 pixels). None of the brain regions had higher metabolism for the control condition (neutral non-task with placebo) than for the cognitive task conditions.

**Figure 3:**
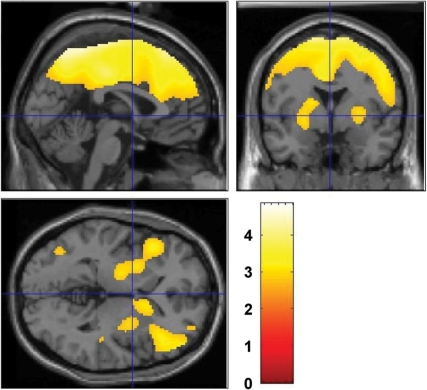
Differences in task activation between placebo (PL) and methylphenidate (MP). SPM results showing the areas that had greater increases in metabolism when the cognitive task was given with placebo versus when it was given with methylphenidate (MP). Comparisons correspond to paired t-tests (p<0.005 uncorrected >100 pixels). None of the brain regions had higher metabolism for the cognitive task when given with MP than with placebo.

The smaller task-related increase in brain consumption of glucose with MP was related to focusing of brain activity. This is shown on the statistical parametric (SPM) analysis, which revealed that the area of significant activation (p<0.001) with the cognitive task was much larger with placebo (67,985 pixels) than with MP (22,632 pixels) (Figure 2). The cognitive task for both conditions (placebo and MP) increased metabolism in left frontal, left parietal, occipital, and cerebellar regions; however when given with placebo, the task additionally increased metabolism in right frontal, right parietal, anterior cingulate, and left thalamic regions (Figure 2). The SPM comparison between the two cognitive task conditions corroborates these differences showing significantly greater activation in frontal, parietal, cingulate, thalamus and hippocampus when the cognitive task was given with placebo than with MP (Figure 3). Independently drawn region of interest analysis revealed similar findings (Table 2).

**Table 2:** Regional glucose metabolism (µg/100 g/min) when subjects were tested during the neutral non-task with placebo (control condition), cognitive task with MP and cognitive task with placebo.

Frontal Cortex	Control Condition		Cognitive task with MP		Cognitive task with PL		Cognitive Task MP v PL	
	Left	Right	Left	Right	Left	Right	Left	Right
Parietal								
BA 5	39.1±7	40.0±7	43.2±7^a^	42.7±8	47.7±8c	47.7±8c	0.001	0.001
BA 7	45.2±7	44.6±8	50.1±9b	50.0±9^a^	55.8±9d	54.4±9d	0.002	0.005
BA 39	44.1±6	43.7±7	49.2±9^a^	46.9±9	52.7±9c	51.1±9c	0.02	0.002
BA 40	44.5±7	44.8±8	50.0±10^a^	49.0±10^a^	53.5±9d	53.4±9d	0.01	0.004
Frontal								
BA 6	44.0±7	44.6±7	50.4±9c	49.9±9^a^	54.7±9d	54.8±9d	0.005	0.003
BA 8	45.1±8	47.5±9	48.4±9	51.0±10	52.9±8c	56.2±9c	0.002	0.003
BA 9	42.4±7	44.3±7	46.6±9^a^	48.4±9^a^	50.1±8c	52.3±8c	0.008	0.007
BA 11	41.6±7	42.3±7	43.4±9	43.5±8	44.0±7	44.1±8	0.64	0.66
Anterior CG								
BA 24	37.0±6	36.9±6	40.1±8	39.9±8	43.9±8c	43.2±8c	0.003	0.02
BA 32	40.9±7	42.8±8	45.1±8^a^	47.6±9^a^	48.5±8c	50.1±9d	0.008	0.02
Temporal								
BA 13	38.2±7	37.52±7	41.8±8	39.9±8	44.5±8b	42.6±7b	0.03	0.02
BA 22	43.1±6	45.4±6	47.8±8^a^	49.1±8	50.8±8c	52.0±9c	0.03	0.03
Occipital								
BA 17	49.4±10	51.2±10	61.0±12d	63.4±10d	64.9±13d	67.6±12d	0.03	0.05
BA 18	48.2±8	47.5±8	55.8±9d	55.5±10d	59.3±10d	59.0±10d	0.02	0.03
BA 19	45.0±7	44.0±7	49.9±9^a^	48.1±8^a^	52.9±9c	51.2±8c	0.03	0.03
Limbic								
Hippocampus	23.4±5	23.6±5	25.3±4	24.0±5	27.6±5^a^	27.0±5^a^	0.04	0.03
Amygdala	27.7±6	26.0±6	30.6±6	28.1±7	33.3±6b	31.9±7^a^	0.006	0.02
Striatum								
Caudate	40.3±8	34.9±8	43.1±10	36.1±7	46.1±10^a^	40.2±9^a^	0.05	0.003
Putamen	42.8±7	40.5±7	45.7±9	43.1±9	49.4±8b	46.7±8b	0.009	0.008
Thalamus								
Medio dorsal	44.5±1	40.9±9	51.4±12b	47.5±10c	54.4±11c	50.0±9c	0.10	0.07
Ventro Lateral	30.7±7	35.6±8	34.2±7^a^	40.7±8c	38.2±8c	43.2±9c	0.002	0.08
Ventro Post Lat	29.7±7	36.6±7	32.8±5^a^	41.6±8b	37.3±7c	44.8±9c	0.004	0.03
Ventro Post Md	41.2±8	44.2±7	46.6±7c	52.5±11d	51.5±9d	56.2±11d	0.01	0.05
Cerebellum	35.1±5	35.0±5	40.1±7c	40.0±7c	41.8±7c	41.4±7c	0.19	0.25

Data corresponds to mean and standard deviation. Subscripts correspond to paired t-test comparisons with respect to the control condition:

a p<0.05, ^b^ p<0.01, ^c^ p<0.005, ^d^ p<0.001. The last column corresponds to the significance level for comparison between the cognitive task when given with MP or when given with placebo (PL).

In contrast to the differences between placebo and MP when given with the cognitive task there were no differences in brain metabolism when MP was given with the neutral non-task condition (whole brain metabolism: 35.8±5 µmol/100 g/min) when compared with the neutral non-task condition when given with placebo (36.6±6 µmol/100 g/min). The SPM analysis corroborated this and revealed no significant differences when the neutral non-task condition was given with placebo (control condition) versus when it was given with MP (data not shown).

## Discussion

This study documents that when MP was given with a cognitive task it markedly attenuated the brain metabolic increases induced by the task and reduced the regions activated by it. The reduction in activation with MP included the parietal cortex, cingulate gyrus and thalamus, which are regions involved in the orienting, executive, and alerting attentional networks respectively[8]. Thus, we interpret our findings to indicate that compared to placebo MP reduced (focused) the use of attentional resources in the human brain that are necessary to achieve similar levels of performance on a task.

These findings are consistent with those of prior imaging studies showing reductions with MP in the increases in cerebral blood flow (CBF) in dorsolateral prefrontal and posterior parietal cortices when healthy controls performed a working memory task[9] and in prefrontal cortex when adults with ADHD performed a task of executive function[10]. However, the MP-related attenuation of CBF increases by the task in these studies was much more restricted (focused to discrete brain regions) than the large and extensive attenuation in whole brain metabolism we report using [^18^F]FDG. Glucose metabolism may offer an advantage because it is a more proximal measure of neuronal activity than CBF[11]. Moreover, regional CBF may become uncoupled from metabolism during stimulation[12], [13].

Synaptic levels of DA and NE, which are increased by MP[5], under physiological conditions act primarily as neuromodulators changing the efficacy of other transmitter signals[14], [15] as a function of ongoing neuronal activity[16]. For example, in striatum, applications of DA decrease the activity of spontaneously active neurons to a greater extent than that of glutamate-stimulated neurons[6]. This increase in glutamate-induced excitation relative to baseline is assumed to improve signal-to-noise neuronal activation[17]. Norepinephrine can also facilitate excitatory transmission by depressing the level of basal activity[18]. The greater decreases in spontaneous neuronal firing (basal activity) than in task relevant neuronal responses from MP's dopaminergic and noradrenergic effects could therefore explain the reduction in the metabolic increases (as well as CBF decreases) induced by the cognitive task. In addition the global effects in metabolism that we observed with MP while performing the task may reflect downstream effects of increasing signal to noise in regions processing the task into regions whose background activity covary with that of regions activated by the task[19].

The dependency of DA and NE effects as a function of the ongoing neuronal activity[17] could explain the differential response to MP we observed across the neutral and cognitive task conditions (i.e., no effect when given with a neutral non-task but attenuation of increases in metabolic activation when given with a cognitive task). Similar results were reported for MP effects on CBF; decreases in task related activation but no changes with the control condition[9]. The task dependency of MP effects is consistent with clinical findings documenting that the effects of stimulant medications are context dependent[20], [21].

It is worth noting that while most individuals showed lower metabolic activation during the cognitive task with MP than with placebo (16 of 23), five subjects showed greater activation with MP than with placebo and 2 subjects did not change. Because only 5 subjects showed an enhancement with MP we did not have sufficient power to assess if there were differences in baseline brain metabolism or in brain activation to the task between the group of subjects in whom MP decreased versus those in whom it enhanced activation. However, the correlation analysis revealed that the difference in activation between MP and placebo during the cognitive task was correlated both with baseline brain metabolism (control condition) and with the brain activation to the task when preceded by placebo. That is, subjects in whom MP produced the largest attenuation in activation to the task were the ones that had lower brain metabolism at baseline but also had the largest brain metabolic increases when the cognitive task was given with placebo. Subjects with minimal activation to the task were the ones in whom MP produced the least change and were also the ones that did not improve performance with MP (assessed by monetary earnings). This is consistent with the notion that those individuals who already have “optimal focusing” of brain resources would show no benefit from MP. The dependency of MP effects to the magnitude of activation to the task (when given with placebo) is also consistent with idea that the effects of MP in a given subjects are rate dependent; that is determined by their baseline level of performance[22], [23].

The correlation analysis between the difference in money between the cognitive task with MP versus with placebo and the differences in metabolic activation between these two conditions was significant in the paracentral lobule (BA 5), dorsal and posterior CG/precuneus (BA 23, 29, 30, 7) and in parietal cortex (BA 39, 40, 7); subjects in whom MP induced the largest attenuation were the ones that made more money with MP than with placebo. The dorsal and posterior CG, the paracentral lobule and the inferior parietal cortices are regions that form part of the default network, which is deactivated when performing a task[24] and activated during mind-wandering (BA 31, 29, 30)[25]. Thus one could speculate that the ability of MP to decrease the activation in the default network and to decrease mind-wandering is one of the mechanisms that accounts for its beneficial effects in subjects in whom it improves performance. However, in individuals in whom the default network is already optimally deactivated during the task, MP may deteriorate performance as was the case for the 4 subjects in our study who made less money with MP than with placebo.

Though it was once assumed that the beneficial effects of stimulant medications (including MP) on individuals with ADHD were paradoxical, studies have demonstrated that the direction of response is the same in healthy individuals without ADHD[26]. This confusion may reflect in part the fact that the responses to stimulant medications are dependent on the initial level of performance; typically performance is improved only when cognitive processing is below optimal, resulting in a non-monotonic (U-shaped) function[27]. Our findings suggest a neural mechanism for this: we postulate that when neuronal resources are widely distributed across brain regions, the action of MP to focus (reduce) regional activation would improve performance on a specific task, whereas the MP-related restriction of regional brain activation when already optimally deployed could impair performance.

The oral dose of MP used in this study (20 mg) is within the range used therapeutically for the treatment of ADHD in adults. The lack of an effect on brain metabolism with the neutral non-task suggests that this dose of MP without a concomitant cognitive activation does not affect brain activity. This is consistent with our prior findings showing that 20 mg of oral MP did not significantly increase DA in the striatum (assessed with PET and [^11^C]raclopride) when given with a neutral non-task whereas it increased it when MP was administered concomitantly with a cognitive task (same numerical calculations task used for the current study)[28]. It is also consistent with prior imaging studies showing minimal changes in regional brain glucose metabolism in ADHD subjects given MP without stimulation[29].

Limitations for this study include the fact that the assessment of brain glucose metabolism with PET and FDG reflects the average activity of the brain over a 30 minute period, which does not allow an assessment of the dynamic changes that may occur during that time period. Our experimental design did not allow us to evaluate the relationship between the inter-subject variability in the brain metabolic responses to MP during the task and an individual's level of performance. In our design, the difficulty of the task was adjusted so that each subject would achieve a constant level of performance (about 80% accuracy), and the adjustments varied across individuals since they depended on each individual's level of ability on the mathematical task as well as his/her ability for the different mathematical operations. Also in this study the amount of money made during the task (a possible indicator of a subject's overall performance) was constrained by the adjustment procedures. In future studies, different designs with more precise measures of performance could be used to evaluate the extent to which the differences between subjects and within subjects in response to MP relate to difference in their performance capacity and how this information can be used to predict response to stimulant medications.

### Summary

This study shows that compared to placebo, an oral dose of MP reduced the brain metabolic increases associated with performance of a cognitive task. Inasmuch as the brain required about 50% less increase in glucose to perform the task at the same level of performance, this provides evidence that one of the mechanisms of action of MP is to focus activation and make the brain more efficient.

Our study of the effects of MP on brain function in healthy adults may contribute to theoretical basis for how and when stimulant drugs may (or may not) enhance attention and performance. To the extent that neuronal resources are non-optimally distributed, reduced task-induced regional activation could result in improved performance. Non-optimal distribution of attentional resources may occur in some individuals (ie., those with ADHD) or in healthy individuals after sleep deprivation. However, if neuronal resources are already optimally deployed, further focusing of neuronal activity could result in stimulant-related deterioration of performance.

## Materials and Methods

### Subjects

Twenty three healthy controls (12 M and 11 F; 32±7 years of age) who responded to an advertisement were studied. Subjects were initially screened by phone and then evaluated at Brookhaven National Laboratory by a physician for exclusion criteria, which included current or past psychiatric disorder (including drug abuse or dependence), neurological disease, significant medical illness, current treatment with medication (including over the counter drugs) and pregnancy. Normal physical examination and laboratory tests were required for entry. Pre-scan urine tests ensured the absence of any psychoactive drugs and of pregnancy in females. Subjects were monetarily compensated for their participation. Written informed consent was obtained in all subjects in accordance with the local Institutional Review Board.

### Scans

PET scans were obtained with a whole-body, high-resolution positron emission tomograph (Siemens/CTI ECAT HR+, with 4.6×4.6×4.2 mm NEMA (National Electrical Manufacturers Association) resolution at center of field of view and 63 slices) in 3D dynamic acquisition mode using [^18^F]FDG. Details about the methods for scanning have been published[30]. Briefly, a 20 minute emission scan was started 35 minutes after injection of 4–6 mCi of [^18^F]FDG. Arterialized blood sampling was used to measure FDG in plasma.

All subjects were scanned 3 times with [^18^F]FDG under the following conditions: 1. Neutral non-task preceded by placebo, which was the “control” condition; 2. Cognitive task preceded by MP; 3. Cognitive task preceded by placebo. In addition, 16 of the 23 subjects underwent a fourth [^18^F]FDG scan conducted with the neutral non-task preceded by MP. Each scan was performed on a separate day and subjects were blinded as to whether they received MP (20 mg po) or placebo. The order of the conditions was balanced across subjects. Venous blood was drawn to quantify plasma concentrations of MP prior to and at 60, 90 and 120 minutes after MP using capillary GC/Mass spectrometry[31]. Plasma concentrations did not differ between conditions and averaged 6.5±2 ng/mL between 90–120 minutes after administration.

### Tasks

For the cognitive task the subjects were first assessed on their numerical abilities using numerical problems grouped into 5 levels of difficulty. The level at which each individual responded correctly to 80% of the problems was selected for the testing procedure. The numerical problems (additions, subtractions, multiplications or divisions) were presented on colored cards (one card per minute). Within individuals, problem difficulty was adjusted dynamically based on each individual's cumulative performance to try to maintain a constant level of performance across individuals and conditions (with a target of 80% accuracy). Correct responses were remunerated by 25 cents to one dollar depending on the difficulty of the question. The total amount made at the end of the session was quantified and used as surrogate for performance. For the neutral non-task, subjects were shown cards with pictures of scenery but were not asked to provide responses nor were they remunerated. The tasks were started 15 minutes prior to radiotracer injection (60 minutes after placebo or MP) and were continued for 45 minutes (Figure 1).

### Image and data Analysis

The data were analyzed both using regions of interest (ROI) and Statistical Parametric Mapping (SPM)[32].

For SPM analysis, the metabolic images were spatially normalized using the template provided in the SPM 99 package and subsequently smoothed with a 16 mm isotropic Gaussian kernel. Paired samples t-tests were performed for the following comparisons: 1. control condition versus cognitive task with MP; 2. Control condition versus cognitive task with placebo; 3. cognitive task with MP versus cognitive task with placebo; and 4. control condition versus neutral non-task with MP (this comparison was for 16 subjects). Significance was set at p<0.001 (uncorrected, >100 voxels) for the comparisons against the control condition and to p<0.005 for the comparisons between the two cognitive task conditions. Statistical maps were overlaid on an MRI structural image.

For ROI analysis, we extracted independently drawn ROI using an automated extraction method that is based on the standard brain template from the Talairach atlas[33]. First, to eliminate variations across individuals' brains, the [^18^F]FDG images were mapped into the Talairach brain using the spatial normalization package in SPM. The inverse mapping procedure was used to extract the Talairach coordinates of all voxels for a given anatomical region using the stereotactic coordinates in the Talairach Daemon database[34], [35]. These anatomically defined ROIs were overlapped voxel-by-voxel onto the SPM normalized PET image.

To compare the metabolic values in the ROI we used a repeated measure ANOVA for the 3 conditions for which we had measures in all subjects (control condition, cognitive task with MP and cognitive task with placebo). Post hoc t-tests were used to assess which conditions differed. In the 16 subjects for whom the neutral non-task with MP condition was obtained we used a paired t-test to compare it with the control condition. Because of the multiplicity of comparisons to protect against type 1 error we set the level of significance to p<0.005; we did not use Bonferroni correction since it assumes independence between measures whereas the regional metabolic measures are not independent from one another. Also we only consider significant findings that were corroborated both by SPM and ROI analysis. We also analyzed the individual responses and used chi-square analysis to compare the number of subjects in whom MP attenuated the metabolic increases to the task (>2.5%) versus the number of subjects in whom MP enhanced them.

Pearson product moment correlations were performed to assess if the differences in brain activation with the cognitive task (placebo versus MP) were associated with: (1) baseline brain metabolism, (2) brain activation when the task was given with placebo, or (3) brain activation when the task was given with MP. We also performed correlation analysis to assess the relationship between the difference in regional activation during the cognitive task when given with placebo versus MP and the difference in the money earned between both conditions (used as surrogate marker of performance).
